# Real-World Case Series of Ravulizumab Use in Patients with Myasthenia Gravis in Romania

**DOI:** 10.3390/brainsci15040350

**Published:** 2025-03-28

**Authors:** Crisanda Vîlciu, Oana Antonia Mihalache, Bogdan Marius Istrate, Mihaela Aftinia Marian, Mirela Ramona Drăghici, Diana Mihaela Petrescu, Adriana Octaviana Dulămea, Daniela Cristina Anghel

**Affiliations:** 1 Department of Neurology, “Carol Davila” University of Medicine and Pharmacy, 020021 Bucharest, Romania; crisanda.vilciu@umfcd.ro (C.V.); mihaela.marian@umfcd.ro (M.A.M.); diana-mihaela.vlad@rez.umfcd.ro (D.M.P.); octaviana.dulamea@umfcd.ro (A.O.D.); daniela.anghel@umfcd.ro (D.C.A.); 2 Department of Neurology, “Fundeni” Clinical Institute, 022328 Bucharest, Romania; mirelaneuro@yahoo.com; 3 Independent Researcher, 3000 Leuven, Belgium; istratem.bogdan@yahoo.com

**Keywords:** myasthenia gravis, ravulizumab, QMG, MG-ADL, MG-QoL15r

## Abstract

**Background and Objectives:** Ravulizumab, a long-acting C5 complement inhibitor, was approved in the US and Europe in 2022 as an add-on therapy for the standard treatment of AChR-positive generalized MG (gMG). We share our real-world experience with adult patients receiving this therapy in Romania. **Materials and Methods:** Six AChR-positive gMG patients received ravulizumab through an Early Access Program (January 2023–October 2024). Patient outcomes were assessed at the therapy start and q8w using Quantitative MG (QMG), MG Activities of Daily Living (MG-ADL), and MG Quality of Life 15-item revised (MG-QoL15r) scales. **Results:** Age at disease onset ranged from 15 to 35 years. Four of the six patients were women. Two patients had gMG severity level of IIa, and four patients of IIb according to the Myasthenia Gravis Foundation of America (MGFA) classification. Five patients experienced rapid and sustained improvements in MG symptoms with MG-ADL score reductions ranged from −3 to −5 at 26 weeks post-ravulizumab start (except for those with a low baseline score: three and one). QMG score dropped in three patients (−2 to 12) during the treatment period, increased in two (+2 and +8), and remained stable in one (zero). Three patients showed sustained improvement in MG symptoms after ≥60 weeks. MG-QoL15r significantly dropped (−22 to −10) throughout the treatment period. One patient experienced ravulizumab-associated adverse events (vomiting, diarrhea, chills) that resolved within 24 h following symptomatic management, two to three episodes of myasthenic exacerbations during treatment, and discontinued it. **Conclusions:** All cases presented here had early-onset AChR antibody-positive, non-thymomatous MG. Despite differences in disease duration and underlying conditions, clinically meaningful and sustained improvements in gMG symptoms, and reduced corticosteroid doses were observed in all patients except one after adding ravulizumab to the treatment plan.

## 1. Introduction

Myasthenia gravis (MG) is a rare autoimmune disorder with an incidence ranging from 0.63 to 2.9 per 100,000 person-years and a prevalence ranging from 11.2 to 36.1 per 100,000 persons in Europe [1]. MG incidence peaks in women aged 20 to 40 years, men after 50 years, and between 60 and 70 years in both sexes. Approximately 10% of cases occur before the age of 18 [2,3].

MG is characterized by fluctuating muscle weakness that affects the ocular, bulbar, axial, and limb muscles. The severity of this weakness varies and is often exacerbated by physical activity [2,4]. Severe cases can progress to life-threatening respiratory failure [4]. Approximately 85% of MG cases are associated with the presence of autoantibodies against acetylcholine receptors (AChR) targeting proteins of the neuromuscular junction (NMJ) of skeletal muscles. Approximately 5% of patients with MG have autoantibodies directed against muscle-specific tyrosine kinase (MuSK), 1–3% have antibodies against lipoprotein receptor-related protein 4 (LRP4), and 10–15% have no detectable antibodies [3,5]. In seronegative patients experiencing active symptoms, diagnosing MG is particularly challenging and may lead to delayed or misdiagnosis [6].

Clinically, ocular weakness is the first symptom of MG in approximately 75% of cases. Up to 80% of patients progress from ocular MG to generalized MG within two years [3]. In MG patients, changes at the level of the thymus gland, such as thymic hyperplasia or the presence of a thymoma, are commonly observed and are believed to contribute to the autoimmune mechanisms underlying the disease. Around 70% of AChR-positive MG patients have thymic hyperplasia, while approximately 10% have a thymoma [7]. Additionally, approximately 25% of MG patients are affected by other autoimmune conditions, with the most frequent being systemic autoimmune diseases such as thyroid disorders, systemic lupus erythematosus, and vitiligo [7]. Neurological autoimmune conditions, including neuromyelitis optica, inflammatory myopathy, and autoimmune encephalitis, are also observed [7].

Even though no curative treatment is currently available for MG, the disease can be effectively managed in most cases [8]. The standard therapeutic management has relied on acetylcholinesterase inhibitors as symptomatic therapy, corticosteroids, and immunosuppressants (e.g., azathioprine, cyclosporine, mycophenolate mofetil, or tacrolimus), which have demonstrated promising efficacy in improving MG-related symptoms in most patients and lowering mortality rates. Thymectomy is recommended for individuals with generalized AChR-positive MG under 50 years of age, or under 65 in certain countries, as well as for those with thymoma-associated MG [9,10]. Plasmapheresis and intravenous immunoglobulins (IVIg) are used as rescue therapies for individuals undergoing a myasthenic crisis or for those who are refractory to standard treatments [11]. However, the conventional immunosuppressants pose a considerable risk of long-term adverse effects that increase MG burden, and up to 15% of MG patients show limited or no response [11,12].

Recent advances in MG management have substantially improved patient outcomes due to the changes in the standard of care and evolving treatment landscape, including the approval of new therapies targeting the underlying pathophysiological pathway of the disease [13]. Among these options, molecular therapies such as B-cell-depleting agents, complement inhibitors, neonatal Fc receptor antagonists, and chimeric antigen receptor T-cell therapies are emerging treatments [14].

Ravulizumab is a long-acting C5 complement inhibitor agent approved in April 2022 in the United States [15] and September 2022 in Europe [16] as an add-on to standard treatment for AChR-positive generalized MG (gMG) based on the pivotal phase three CHAMPION-MG trial results [17]. Belgian, German, and Nordic European countries’ guidelines have already been updated, and ravulizumab has been recommended as an add-on treatment in adult patients diagnosed with AChR-positive gMG [3,18,19].

In Romania, ravulizumab has been accessible to AChR-positive MG since early 2023 via the Early Access Program (EAP). To provide insights into using ravulizumab in real-world settings, we share our experience with a small series of adult patients with AChR-positive MG who received ravulizumab as part of the EAP.

## 2. Materials and Methods

Between January 2023 and October 2024, six patients with AChR-positive gMG received ravulizumab as an add-on therapy. Patient information was collected at their most recent visit within the study period when they were admitted for disease monitoring and administration of the ravulizumab dose.

Patient outcomes were measured by using the Quantitative Myasthenia Gravis (QMG), Myasthenia Gravis Activities of Daily Living (MG-ADL), and Myasthenia Gravis Quality of Life 15-item revised (MG-QoL15r) scales. Higher scores indicate more reported symptoms or more severe disease.

The treating physicians assessed QMG and functional MG disease severity, including ocular, facial/oropharyngeal, and non-facial symptoms. The total QMG score ranges between 0 and 39, with higher scores suggesting greater disease severity [20,21]. A 3-point change in QMG is regarded as clinically meaningful improvement and a 5-point change is regarded as “responder” status [22,23].

MG-ADL was administered by the physician or trained clinic personnel. The patient-reported outcomes included eight items: breathing, talking, chewing, swallowing, ability to brush teeth or comb hair, ability to rise from a chair, double vision, and eyelid droop. The total MG-ADL score ranges between 0 and 24, with higher scores suggesting greater severity of symptoms. A 2-point improvement in MG-ADL score is a threshold that suggests minimal clinical improvement and a 3-point improvement in MG-ADL is considered as “responder” status [23,24].

The MG-QoL15r questionnaire allowed physicians to assess the impact of disease on patients’ emotions, physical health, self-care, and social life. The total MG-QoL15r score ranges between 0 and 30. Higher scores indicate a poorer quality of life [25].

All patients received the anti-meningococcal vaccine, which targets various serotypes of *Neisseria meningitidis*, approximately one month prior to the initiation of ravulizumab treatment. The vaccination regimen was completed with the entire course, including the conjugate tetravalent vaccine against meningococci serogroups A, C, Y, and W and the vaccine against meningococcal serogroup B.

All cases were comprehensively analyzed to summarize key patient characteristics, describe treatment patterns, and highlight relevant clinical insights.

## 3. Results

Table 1 includes a summary of patient and AChR-positive MG disease characteristics, as well as treatments received before ravulizumab inclusion in their therapeutic plan.

### 3.1. Case 1

A 20-year-old female (height: 168 cm, weight: 64 kg) with a confirmed diagnosis of AChR-positive MG first presented in 2020 at the age of 16 with effort-dependent muscle weakness initially affecting the lower limbs, which later progressed to the upper limbs. She experienced recurrent severe exacerbations despite treatment with escalating doses of prednisone (ranging from 5 mg to 60 mg daily) in combination with pyridostigmine (60 mg, four to six tablets daily). A detailed summary of her medical history is provided in Table 1.

**Figure 1:**
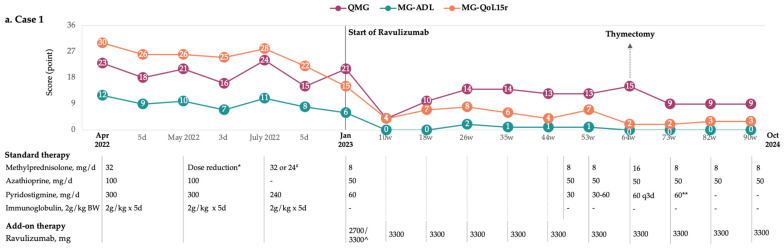

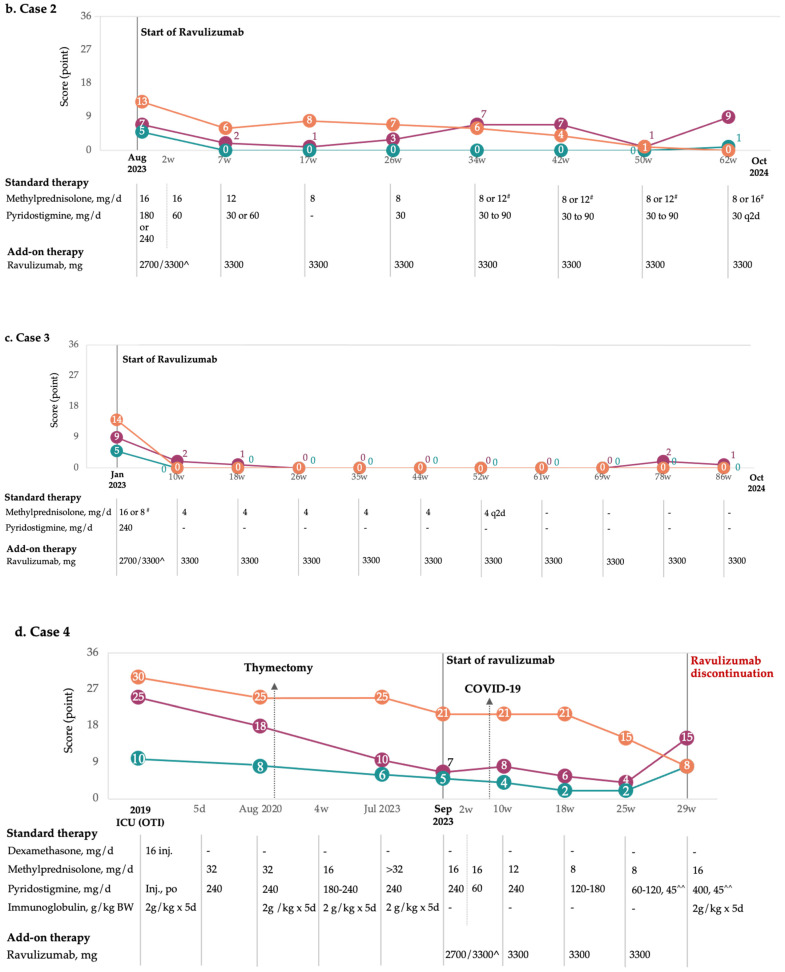

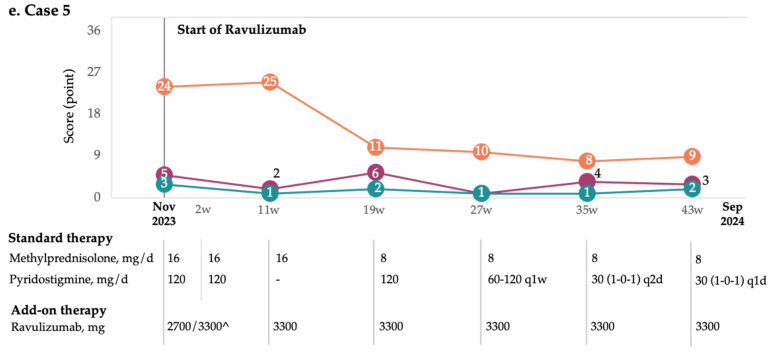

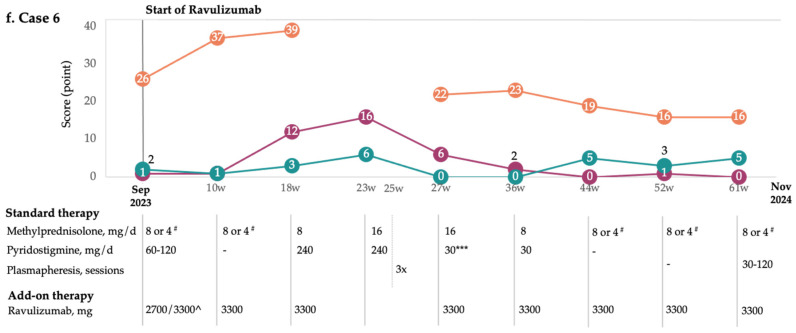
Treatment and clinical course of patients with myasthenia gravis: (**a**) Case 1; (**b**) Case 2; (**c**) Case 3; (**d**) Case 4; (**e**) Case 5; (**f**) Case 6. * four mg decrease q2d for one month; ^#^ alternatively; ** one-month break; *** when required, during menstruation period; ^ loading dose/first maintenance dose administered after two weeks; ^^ extended-response formulation. Abbreviations: d, days; BW, body weight; D, dose; w, weeks after ravulizumab treatment start; QMG, quantitative myasthenia gravis score; MG-ADL, MG activities of daily living score; MG-QoL15r, MG quality of life revised 15 score; OTI, orotracheal intubation; qxd, every x days; qxw, every x weeks. Notes: The anti-meningococcal vaccine was administered around one month before ravulizumab treatment initiation in all patients. In patient 6, the unrevised version of the MG-QoL15 was used due to its previous use in clinical practice and the availability of previous assessments based on this version.

Her daily activities were significantly impaired due to persistent muscle weakness and fatigue. Additionally, prolonged high-dose prednisone therapy resulted in corticosteroid-induced myopathy and dyslipidemia. In April 2022, at the age of 18, she was referred to our clinic with a moderate-to-severe weakness affecting the proximal upper and lower limbs, trunk, and cervical muscles. She also exhibited mild-to-moderate bulbar dysfunction, asymmetric ptosis in both eyes, and diplopia. Electromyography (EMG) revealed a 30% decrement in the proximal upper limbs, with clinical evaluations yielding a QMG score of 23, MG-ADL of 12, and MG-QoL15r of 30 (Figure 1a). Prednisone was replaced by methylprednisolone in an attempt to provide faster and more effective symptom relief. Additionally, azathioprine, along with repeated cycles of IVIg, was introduced for the treatment of gMG. Despite symptom improvement with immunoglobulin treatment, the patient experienced two severe exacerbations within 6 months; in July 2022, she presented with a generalized exacerbation, primarily characterized by significant limb weakness (Figure 1a). EMG demonstrated a myasthenic decrement at the facial level and a chronic mild, asymmetric involvement of all four limbs with myogenic features. Clinical assessments recorded a QMG score of 24 and MG-ADL of 11. Due to worsening symptoms, a new cycle of IVIg was initiated; however, the clinical improvement observed outside the exacerbation periods remained minimal (Figure 1a).

In January 2023, ravulizumab therapy was initiated under the EAP program. Two months after the first maintenance dose, the myasthenic deficit was substantially reduced (QMG: four, MG-ADL: zero), and the quality of life was improved (MG-QoL15r: four). A slight increase in arm and leg muscle weakness was observed 18–24 weeks after treatment initiation, with no identifiable cause. However, this weakness was detectable only through objective assessments as the patient did not subjectively perceive it, it did not impact her daily activities, and she did not feel the need to administer anticholinesterase therapy. A chest CT revealed increased thymus dimensions, and the patient underwent thymectomy. Histopathological examination confirmed the presence of a thymolipoma with a thymic cyst. The postoperative outcome was favorable. During consecutive follow-up visits, the patient demonstrated stable disease, allowing for a reduction in the methylprednisolone dose and the gradual discontinuation of pyridostigmine, as it was no longer needed due to symptom improvement. A mild myasthenic deficit in the limbs was observed, likely due to these adjustments. Despite this deficit, the patient reported no worsening symptoms, and her ability to perform daily activities remained unaffected (Figure 1a). During approximately 1.7 years of treatment, no adverse reactions related to ravulizumab or disease exacerbations were observed. Currently, she is continuing the ravulizumab therapy, combined with a reduced dose of methylprednisolone, azathioprine, and pyridostigmine (when required). Quarterly complete blood count (CBC), aspartate transaminase (AST), and alanine transaminase (ALT) monitoring were recommended. A six-month supporting treatment, including calcium 600 mg (q2d), vitamin C 1 g (1-0-0 q1d), and vitamin D3 2000 international units (IU) (1-0-0 q1d), was recommended. The next dose of ravulizumab was scheduled eight weeks after the last one.

### 3.2. Case 2

A 30-year-old male (height: 170 cm, weight 65 kg) diagnosed with AChR-positive MG in 2007 (the initial symptoms were bulbar, manifesting as dysphagia for liquids, dysphonia worsened by exertion, and difficulty chewing, subsequently, the myasthenic weakness progressed, affecting the facial muscles and extending to the limbs) was admitted to our department for treatment. Detailed information on his medical history is provided in Table 1. For 16 years, he was treated with high doses of cortisone and pyridostigmine, but he experienced frequent episodes of symptom relapse. In April 2023, he presented with a severe exacerbation of generalized symptoms. His treatment regimen included oral methylprednisolone at a dose of 24 mg/day, neostigmine 0.5 mg/mL (2 vials q4h), and IVIg 2 g/kg body weight. He was ineligible for azathioprine due to intolerance, which manifested as leukopenia and pityriasis versicolor.

Owing to his poor response to previous treatments, in August 2023, ravulizumab was introduced to his treatment plan as part of the EAP. Following ravulizumab initiation, methylprednisolone and pyridostigmine doses were substantially reduced and adjusted according to the patient’s needs (Figure 1b). A substantial improvement in the MG-ADL (from 5 to 0) was observed throughout the follow-up period, while the MG-QOL-15 score decreased progressively (from 13 to 0). QMG score dropped from 7 to 3 after the fourth dose of ravulizumab but increased to 6.5 after the sixth dose and 8.5 after the eighth (Figure 1b). However, the patient reported that facial and spinal muscle weakness substantially improved during ravulizumab treatment compared to the previous period. Additionally, although a mild reduction in exercise tolerance, particularly during sustained effort, was reported before the sixth dose, the disease stabilized afterward with enhanced effort tolerance. No ravulizumab-related adverse reactions and disease exacerbations were recorded during approximately 1.2 years of treatment.

Currently, he is continuing the ravulizumab therapy. Monthly CBC, AST, and ALT monitoring were recommended at discharge. Additionally, supporting treatment including mycophenolate mofetil 500 mg (1-0-0 q1d for one week, then 1-0-1 q2d), calcium 600 mg (q2d), vitamin C 1 g (1-0-0 q1d), vitamin D3 2000 IU (1-0-0 q1d), and potassium 99 mg (0-0-1 q1d) were recommended. The next dose of ravulizumab was scheduled eight weeks after the last one.

### 3.3. Case 3

A 31-year-old male (height: 177 cm; weight: 82 kg) diagnosed with AChR-positive gMG in 2019 was admitted to our department for treatment continuation. Details on the disease history are shown in Table 1. For three years, he experienced three to four disease exacerbations yearly with moderate to severe myasthenic deficits characterized by effort-dependent limb weakness, resulting in residual weakness and poor motor performance between exacerbations, which significantly affected his day-to-day activities and led to anxiety and depression, impacting his quality of life. Outside of exacerbation periods, the patient was on long-term treatment with methylprednisolone 16 mg (po daily) and pyridostigmine 180 mg (daily), while during exacerbations, methylprednisolone dose was increased to 32 mg daily and pyridostigmine to 240–300 mg.

In January 2023, ravulizumab was added to his treatment plan as part of EAP. Concomitantly, he received venlafaxine 75 mg daily to treat the major depression disorder. The patient reported significant improvement in ocular symptoms and limb weakness. Pyridostigmine was discontinued two months after the initiation of ravulizumab, as it was no longer needed due to symptom resolution.

Methylprednisolone doses were substantially reduced in the first year and then discontinued as per patient self-judgment (Figure 1c). The improvement of symptoms continued with increased exercise tolerance and no requirement for pyridostigmine. At 35 weeks after the initiation of ravulizumab, venlafaxine treatment was discontinued. Moreover, one year after ravulizumab started, pharmacological remission of MG was observed (QMG: zero points, MG-ADL: zero, MG-QoL15r: zero) (Figure 1c). No ravulizumab-related adverse reactions or disease exacerbations were recorded during approximately 1.6 years of treatment.

At discharge, the following treatment plan was recommended: pyridostigmine 30–240 mg as needed, calcium lactic 500 mg (q1d), vitamin C 1 g (1-0-0 q1d), and vitamin D3 2000 IU (1-0-0 q1d). The next dose of ravulizumab was scheduled eight weeks after the last one.

### 3.4. Case 4

A 26.8-year-old female (height: 160 cm; weight: 61 kg) diagnosed with AChR-positive gMG in 2019 was admitted to our department for the continuation of treatment. Her disease-specific symptoms started in 2016 with dysphonia, which progressively worsened to severe hypophonia, swallowing disorders, and muscle weakness, predominantly in the proximal limbs. An MG diagnosis was confirmed in 2019 following a severe exacerbation that required orotracheal intubation (OTI) with mechanical ventilation and thymectomy was performed in 2020. Other disease characteristics are included in Table 1. For approximately 2.5 years after diagnosis, she experienced severe disease exacerbations, including episodes of dysphonia, dysphagia, generalized muscle weakness, and reduced respiratory tolerance to exercise. She received four cycles of IVIg alongside high doses of pyridostigmine and glucocorticoids. Although these therapeutic interventions determined transient symptomatic relief, the patients’ MG-ADL score dropped 5 points (from 10 to 5), but the MG-QoL15r score remained elevated (from 30 to 21), indicating a substantial impact of the disease on quality of life (Figure 1d). No immunosuppressive therapy was initiated during this period, as the patient declined further treatment. Additionally, glucocorticoid treatments led to complications, such as anxiety disorders, insomnia, and recurrent oral candidiasis.

Ravulizumab treatment course was started in September 2023. After the loading dose and the first maintenance dose, the patient experienced significant improvement in myasthenic symptoms, but shortly thereafter, bulbar and facial symptoms worsened during a concurrent COVID-19 disease, necessitating an increase in the dose of methylprednisolone. Before the third ravulizumab dose, QMG scored 8 points, MG-ADL scored 4 points, and MG-QoL15r 21 points. Post-administration, the patient experienced ravulizumab-related vomiting, diarrhea, and chills. She was admitted to the emergency department and received symptomatic treatment, with symptoms resolving within approximately 24 h. One month after the fourth maintenance dose of ravulizumab, the patient presented with a moderate-to-severe generalized myasthenic deficit with bulbar involvement (dysphonia at rest worsened by effort, moderate facial weakness, moderate limb and cervical muscle deficits, and reduced respiratory tolerance to minimal effort) despite a gradual increase in methylprednisolone and pyridostigmine doses (QMG: 15, MG-ADL: 8; MG-QoL15r: 8). A new cycle of IVIg 2 g/kg body weight was administered, resulting in a slight improvement in the myasthenic deficit (Figure 1d). Given that the patient experienced frequent relapses (every two to three months) while on ravulizumab treatment, along with increased corticosteroid requirements, ravulizumab therapy was discontinued.

The following treatment plan was recommended: methylprednisolone 32 mg, q1d for two weeks, followed by a gradual decrease to 16 mg q1d, pyridostigmine 60–240 mg daily as needed; calcium 600 mg (q1d), vitamin C 1 g (1-0-0 q1d), vitamin D3 2000 IU (1-0-0 q1d), potassium 99 mg (0-0-1 q1d), spironolactone 25 mg (q2d), and azathioprine 50 mg in the first month, followed by 100 mg q1d. Monthly CBC, AST, and ALT monitoring during the first three months and then every three months were also recommended.

### 3.5. Case 5

A 45.6-year-old woman (height: 167 cm; weight: 60 kg) diagnosed with AChR-positive gMG in 1996, with initial presentation characterized by exertion-induced dysphonia, followed by the progressive onset of limb muscle weakness, was admitted to our department for treatment continuation. Details on the disease history are shown in Table 1. For 27 years, she experienced 3–4 severe exacerbations yearly that required treatment adjustment with high doses of methylprednisolone and pyridostigmine. During this period, four of the severe myasthenic crises (QMG up to 34, MG-ADL up to 20) required OTI with mechanical ventilation. These frequent and severe disease exacerbations significantly affected her day-to-day activities and led to anxiety and depression, impacting her quality of life. Outside of exacerbation periods, she underwent long-term treatment with methylprednisolone 32 mg (po daily) and pyridostigmine 300–360 mg (po daily, adjusted as needed). During exacerbations, high doses of methylprednisolone (up to 64 mg daily) or dexamethasone 8 mg (daily injection), cyclophosphamide alongside pyridostigmine 0.5 mg/mL (2 vials IM q4h), and five plasmapheresis sessions or IVIg were administered. These treatments led to multiple complications (Table 1). She was also diagnosed with sinus tachycardia, chronic autoimmune thyroiditis, chronic venous insufficiency, hepatic steatosis, systemic atherosclerosis, coronary artery disease, and hypertension. These associated conditions complicated MG’s treatment, requiring a multidisciplinary approach.

In November 2023, ravulizumab was added to her treatment plan as part of EAP. Her concomitant medications included atorvastatin 20 mg (q1d), fenofibrate 160 mg (q1d), betaxolol 10 mg (q1d), pentoxifylline extended-release 800 mg (q1d), alfacalcidol 1 mg (q1d), and metformin 500 mg (q1d). During the ravulizumab treatment, the patient reported a progressive improvement in her myasthenic deficit, with a substantial increase in exercise tolerance. After receiving the second maintenance dose of ravulizumab, the patient experienced transient muscle weakness in the limbs, likely attributed to physical overexertion. This was resolved promptly with an increase in corticosteroid dosage. Scores on assessment scales showed significant improvement, especially in the MG-QoL15r, which decreased from 24 to 9 from ravulizumab treatment initiation to one year after (Figure 1e). Ravulizumab addition also allowed for significant MG treatment adjustments. The patient successfully reduced the methylprednisolone dose from 16 to 8 mg daily and decreased pyridostigmine from 120 to 60 mg daily (Figure 1e). No adverse reactions related to ravulizumab were observed during approximately one year of treatment.

After the last ravulizumab dose, the following six-month treatment plan was recommended: methylprednisolone 8 mg (1-0-0 q1d), pyridostigmine 60–240 mg (when needed), pantoprazole 20 mg (q1d when required), calcium 600 mg (q1d), vitamin C 1 g (q1d), vitamin E 100 mg (15 days monthly), trazodone 50 mg (when needed), betaxolol 10 mg (1-0-1 q1d), clopidogrel 75 mg (0-1-0 q1d), atorvastatin 20 mg (0-0-1 q1d), sulodexide (1-0-1 q1d), pentoxifylline extended-release 400 mg (1-0-1 q1d), fenofibrate 160 mg (0-1-0 q1d), alfacalcidol 1 µg (q1d), metformin 500 mg (0-0-1 q1d), and alendronic acid 70 mg (q1w). The next dose of ravulizumab was scheduled eight weeks after the last one.

### 3.6. Case 6

A 38.7-year-old woman (height: 163 cm; weight: 61 kg) diagnosed with AChR-positive gMG in 2021 (the patient’s initial presentation was characterized by exertion-induced dysphonia and swallowing difficulties, followed by the subsequent development of limb muscle weakness) was admitted to our department for treatment continuation. Details on the disease history are shown in Table 1. For two years, she experienced four myasthenic exacerbations. The first one occurred in August 2021, when she developed moderate ocular and bulbar symptoms (QMG: 13 points, MG-ADL: 8, MG-QoL15: 24), which were treated with pyridostigmine (240 mg daily), corticosteroids, and IVIg (2 g/kg), but only partial symptom control was achieved. In the same year, she underwent surgery for a benign retroperitoneal tumor located in the left infrarenal region. By December 2021, her condition had significantly deteriorated, leading to a severe myasthenic crisis that required OTI with mechanical ventilation (QMG: 21, MG-ADL: 12, MG-QoL15: 26). During this period, she developed stress-induced cardiomyopathy (Takotsubo), characterized by severe left ventricular dysfunction (LVEF 30%) and extensive hypokinesia of the apical and mid-cardiac segments, as confirmed by coronary computed tomography angiography. Dexamethasone (8 mg), neostigmine injections (0.5 mg/mL q4h), and IVIg (2 g/kg) were administered to stabilize her condition. Still, she experienced complications such as allergic reactions, hypertension, tachycardia, thrombocytopenia, and urinary tract infections. Following this crisis, the patient exhibited persistent severe myopathic weakness in the lower limbs. The etiology was multifactorial, attributed to both prolonged high-dose corticosteroid therapy and critical illness myopathy. Another severe bulbar symptom exacerbation occurred in April-May 2022 (QMG: 14, MG-ADL: 5), and high doses of methylprednisolone (32 mg daily), neostigmine 0.5 mg/mL q6h (changed to pyridostigmine 240 mg), and IVIg (2 g/kg body weight) produced only partial improvement. Thymectomy was performed in June 2022. Even so, a myasthenic crisis with respiratory failure requiring admission to the intensive care unit and non-invasive ventilation with continuous positive airway pressure occurred in November 2022; her treatment was adjusted to dexamethasone, neostigmine, IVIg, and azathioprine. In March 2023, she was diagnosed with osteoporosis and spondylodiscitis, for which she received targeted antibiotic therapy. In this infectious context of spondylodiscitis, the treatment with azathioprine was interrupted to minimize the risk of further complications related to immunosuppression. In May 2023, she underwent the removal of a mammary implant due to rupture and subsequent infection, followed by reimplantation in July 2023. In June 2023, she experienced another severe bulbar relapse (QMG: 17, MG-ADL: 8) that required treatment adjustment to methylprednisolone (32 mg daily), neostigmine 0.5 mg/mL q6h, pyridostigmine 240 mg, and three sessions of plasmapheresis.

In September 2023, ravulizumab was added to her treatment plan. After the first three doses, a significant improvement was observed. However, two weeks after the fourth dose, she experienced a progressive worsening of the generalized myasthenic deficit, predominantly affecting limb muscle strength, requiring three plasmapheresis sessions. After this episode, she steadily improved her myasthenic deficit, and her symptoms remained stable (Figure 1f). No adverse reactions related to ravulizumab were recorded during approximately 1.2 years of treatment.

After the last ravulizumab dose, the following treatment plan was recommended: methylprednisolone 8 or 4 mg (q1d), pyridostigmine 60–240 mg (when needed), calcium 600 mg (1-0-1 q2d), vitamin C 1 g (1-0-0 q1d), vitamin D 2000 UI (q1d), acetylsalicylic acid 75 mg (q1d), and other supportive treatments.

## 4. Discussion

Anti-AChR antibodies are predominantly immunoglobulin (Ig)G1 and IgG3 that contribute to gMG by activating the classical complement cascade upon binding to the AChR. The complement system activation leads to the cleavage of complement C5 into C5a and C5b [13,26]. While C5a possesses pro-inflammatory properties, C5b is associated with other complement proteins and forms the terminal membrane attack complex (MAC). The deposition of the MAC on the NMJ results in the deterioration of the postsynaptic membrane, ultimately leading to a reduction in the number of functional AChRs, thus, compromising the neuromuscular transmission safety factor [26]. As a result, transmission at the NMJ level fails to produce reliable muscle activation, leading to weakness characterized by effort-dependent and variable skeletal muscle involvement, accompanied by fatigue [13,26]. Inhibition of terminal complement activation can prevent MAC formation and NMJ destruction in anti-AChR-positive gMG patients [26].

Ravulizumab is a C5 inhibitor derived from eculizumab and engineered for an extended half-life, allowing therapeutic serum concentrations for over eight weeks. It features four amino acid substitutions: two to prevent target-mediated clearance and two to enhance half-life by increasing affinity for human neonatal Fc receptor (FcRn), thereby improving antibody recycling. These modifications ensure sustained inhibition of terminal complement activity. Unlike FcRn inhibitors, such as efgartigimod, ravulizumab binds to and is recycled through the FcRn pathway without altering FcRn’s overall function [26]. Ravulizumab and eculizumab have both demonstrated significant clinical improvements in treating generalized myasthenia gravis (gMG), as evidenced by data from the *CHAMPION-MG* and *REGAIN* studies [27,28]. Although both medications demonstrate significant efficacy, ravulizumab has the benefit of prolonged dose intervals, hence alleviating the treatment load and improving patient convenience relative to eculizumab, which requires more frequent administration. Moreover, a recent report highlights the steroid-sparing effect and superior Quantitative Myasthenia Gravis (QMG) response observed in eculizumab-treated patients. [29]. These findings underscore ravulizumab as a valuable alternative to eculizumab, offering comparable efficacy with enhanced convenience for real-world application in gMG treatment.

We report six cases of patients with AChR-positive gMG of IIa or IIb severity levels treated with ravulizumab in real-world settings. Five of the six patients experienced rapid and sustained improvements in myasthenic symptoms, as measured by both patient-reported (MG-ADL) and clinician-rated outcomes (QMG). At 26 weeks following the initiation of ravulizumab, MG-ADL score reductions ranged from −3 to −5 in most patients, surpassing the two-point threshold for minimum clinically significant difference [24]. It is important to note that two cases demonstrated only minimal improvement: case 5, likely due to a low baseline score of three, and case 6, due to a baseline score of one. Despite having low baseline scores at the initiation of ravulizumab treatment, cases 5 and 6 had frequent exacerbations and myasthenic crises prior to treatment initiation. Both patients had a history of multiple treatment escalations and required interventions such as plasmapheresis and IVIg during crises

Furthermore, sustained improvement in myasthenia symptoms was observed in three patients with follow-up periods longer than 60 weeks. Specifically, case 1 achieved a −6 reduction in MG-ADL score at 60 and 90 weeks post-treatment initiation, case 2 showed a −4 reduction at 62 weeks, case 3 maintained a −5 reduction at 60 and 86 weeks. These real-world observations support the data from the CHAMPION-MG study, which reported a mean improvement in MG-ADL score from baseline to 26 weeks of −3.1 and of −4 to 60 weeks [17,27].

QMG score reductions varied with disease severity. Case 1 showed a significant decrease of 7 points (from 21 to 14) at 26 weeks after ravulizumab initiation, followed by a drop of −6 (from 15 at 64 weeks, when the patient underwent thymectomy, to 9 at 90 weeks). The improvement in myasthenic deficits achieved with ravulizumab allowed the patient to undergo surgery without the risk of a myasthenic crisis. This patient was the only one who underwent thymectomy while receiving ravulizumab therapy. A notable reduction (−9) was recorded in case 3 (from nine to zero) throughout the follow-up period. Case 5’s score dropped by four (from five to one) at 26 weeks but increased slightly (+2) at 43 weeks. The slight increase in the QMG score for this patient may be attributed to lifestyle changes, including increased physical activity and greater professional and social engagement.

Compared to the CHAMPION-MG study, which reported QMG reductions of −2.8 at 26 weeks and −4.1 at 60 weeks, most patients demonstrated more significant reductions, likely due to baseline disease severity [17]. Existing evidence suggests that the minimal important difference (MID) is higher in patients with severe MG and higher baseline QMG scores (QMG > 16; MID was calculated as 3), than in patients with mild to moderate MG (QMG ≤ 16, MID was calculated as 2) [22].

MG-QoL15r significantly decreased in all patients during the follow-up period (range: −23 to −10), superseding data observed in clinical trials [17,27]. These patients could return to their daily activities, indicating the significant progress achieved in controlling MG and enhancing their quality of life.

In addition to the improvements observed in MG-ADL scores, it is essential to note that all patients reached a patient-acceptable symptom state, defined as an MG-ADL score of two or lower. This further supports the reported efficacy of ravulizumab in treating MG, demonstrating that patients experienced clinical improvement and achieved a level of symptom control that was considered acceptable to them.

One patient (case 4) discontinued ravulizumab after receiving the loading dose and four maintenance doses. During treatment, the patient’s MG-ADL score improved by three points (from five to two) at 25 weeks but increased by six points (from two to eight) following a severe myasthenic crisis one month after the fourth maintenance dose. Similarly, the QMG score decreased by 2.5 points (from 6.5 to 4) at 25 weeks but rose by 11 points (from 4 to 15) at 29 weeks. MG-QoL15r score was decreased by 6 points (from 21 to 15) at 25 weeks. However, due to this severe exacerbation, the patient and physician mutually decided to discontinue ravulizumab treatment. In this case, the treatment with ravulizumab did not influence the evolution of the disease; the patient continued to experience a significant myasthenic deficit with frequent exacerbations. Possible hypotheses for this unsatisfactory response to treatment include the development of antibodies against ravulizumab, leading to its neutralization (the determination of anti-ravulizumab antibodies could not be performed); the persistence of the myasthenic deficit due to external factors (physical overload, stress); the presence of other autoantibodies, or underlying immune mechanisms and the patient might also have a form of the disease that is refractory to this therapy. More research is needed to better understand why some patients do not respond to treatment, particularly by looking at how complement activation and antibody profiles play a role. This will help guide more informed treatment decisions and improve care for patients with refractory forms of gMG. Despite differences in age at treatment initiation and the length of time the disease had been present, all patients in our study were diagnosed with early-onset MG. The patients in this cohort had a range of disease onset ages from 15 to 35 years, which reflects the broad spectrum of when MG can first appear. Although the patients had varying disease durations, from just a few years to over two decades, all of them experienced significant impairments, including frequent exacerbations that required more intensive treatments. Notwithstanding these differences in how long they had lived with the disease, all patients saw substantial improvements after starting ravulizumab therapy. This suggests that even those with long-standing diseases can benefit from complement inhibition.

Interestingly, the age at onset and the length of the disease did not significantly affect how well patients responded to ravulizumab. Most patients experienced clinically meaningful improvements in symptom severity, reduced corticosteroid use, and improved overall quality of life. These findings support the use of ravulizumab as an effective adjunct therapy for early-onset myasthenia gravis, regardless of disease duration or age at onset. A recent report showed that ravulizumab was also effective when administered in myasthenic crisis in an AChR-positive gMG patient. This crisis was characterized by respiratory insufficiency and dysphagia that required intensive care. Following ravulizumab, an almost complete resolution of neuromuscular symptoms was recorded [30]. In our group of patients, no evolutions with ravulizumab administration during refractory myasthenic crises were recorded.

No adverse events related to ravulizumab or disease exacerbations requiring additional rescue treatments were reported in five of the six patients in our case series. One patient experienced adverse events associated with ravulizumab, including vomiting, diarrhea, and chills, which resolved within 24 h following symptomatic management. The occurrence of severe diarrhea and nausea was also reported at similar rates among MG patients in the CHAMPION-MG trial, regardless of whether they received ravulizumab treatment or a placebo [17]. Given that our patient also experienced two to three episodes of myasthenic exacerbations during treatment, the patient discontinued ravulizumab therapy and required a rescue cycle of IVIg. It is noteworthy that in our patient, MG had significantly worsened three years before initiating ravulizumab, with episodes refractory to standard treatments. Moreover, in other patients who continued ravulizumab treatment, a reduction in the doses of standard oral medications for MG was observed during a follow-up period of up to 1.7 years. The doses of cortisone and acetylcholinesterase inhibitors were adjusted individually based on the clinical response and severity of symptoms. Corticosteroid therapy was gradually tapered after the disease stabilized, aiming for the minimum maintenance dose on an alternating schedule.

To ensure patient safety, meningococcal vaccination should be performed at least two weeks before administering the first dose of ravulizumab. Patients who start ravulizumab treatment less than two weeks after receiving a meningococcal vaccine must receive treatment with appropriate prophylactic antibiotics for two weeks post-vaccination [11].

The optimal MG treatment plan should consider the underlying mechanisms, clinical indications, risks and benefits, treatment costs, and the patient’s preferences [13]. As also observed from our case series, real-world treatment experiences differ from data reported in clinical trials due to individual patient factors. EAP implementation is important because life-saving treatments are offered to patients with limited options who are not eligible for enrolment in clinical trials. In addition, real-world safety data and insights into therapy use across diverse patient populations should be generated shortly after approval of a new therapeutic agent, to promote acceptance and uptake among healthcare providers and patients [25].

## 5. Conclusions

To our knowledge, this is the first case series that provides insights into AChR-positive gMG patient outcomes following ravulizumab use as add-on therapy in real-world settings in Romania as part of the EAP program.

Clinically meaningful and sustained improvements in MG symptoms were observed after adding ravulizumab to the treatment plan in all but one patient. Given the complexity of the disease, its chronic nature, and the unpredictable occurrence of myasthenic crises, as well as varying patient responses to standard treatments, an individualized treatment approach tailored to patient-specific factors such as age, the presence of disease-related complications, or other comorbidities is essential for achieving MG disease control and improving patient outcomes and quality of life.

A limitation of this study is the small sample size, which limits the generalizability and statistical power of the findings. With only six patients included, all of whom had early-onset gMG, the results may not accurately reflect the broader population of gMG patients, particularly those with late-onset disease. More extensive studies, including a more diverse patient population, are needed to validate these findings and provide more robust data.

## Figures and Tables

**Table 1:** Clinical characteristics of patients with confirmed gMG and treatments used previous to ravulizumab addition.

Case No.	Sex (F/M)	Age (Years) at Onset	Year of Onset	Diagnosis	gMG Type *	Treatment	EMG	Antibodies (Value)	Neostigmine Test	Thymectomy (Year, Histological Finding)	Related Conditions
1.	F	16	2020	2020	IIa	Immunoglobulin High prednisone doses Methylprednisolone Azathioprine Pyridostigmine	Pathological	AChR (2022: 85 nmol/L 2023: 151 nmol/L)	Positive	Yes (2024: thymolipoma with a thymic cyst)	Cortisone-induced myopathy and dyslipidemia
2.	M	15	2007	2007	IIb	Cortisone Pyridostigmine	Pathological	AChR (2007: 17 nmol/L)	Not performed	Yes (2008: thymc hyperplasia)	Cortisone-induced myopathy, dyslipidemia, and osteopenia
3.	M	32	2018	2019	IIa	Methylprednisolone Pyridostigmine Venlafaxine	Pathological	AChR (2019: 8.3 nmol/L 2024: 127 nmol/L)	Positive	Yes (2020, hypertrophic thymus)	Cortisone-induced myopathy and disuse myopathy, obesity, osteoporosis, erectile dysfunction, major depression
4.	F	20	2016	2019	IIb	Dexamethasone Methylprednisolone Pyridostigmine Immunoglobulin OTI	Pathological	AChR (2018: negative 2019: 4.2 nmol/L)	Not performed	Yes (2020, thymic hyperplasia: 7.5 mm/7.3 mm)	Cortisone-induced anxiety disorders, insomnia, and recurrent oral candidiasis
5.	F	17	1996	1996	IIb	Methylprednisolone Dexamethasone Cyclophosphamide Pyridostigmine Plasmapheresis Immunoglobulins OTI	Not performed	AChR (1996: 7 nmol/L)	Positive	Yes (1997: thymic hyperplasia)	Cortisone-induced severe osteoporosis, spontaneous fractures, osteonecrosis, bilateral cataracts, Cushing’s syndrome, type II diabetes, anxiety, sinus tachycardia, chronic autoimmune thyroiditis, systemic atherosclerosis, coronary artery disease, hypertension, chronic venous insufficiency, hepatic steatosis and depression
6.	F	35	2021	2021	IIb	Methylprednisolone Dexamethasone Pyridostigmine Neostigmine Plasmapheresis Immunoglobulins Azathioprine OTI	Pathological	AChR (2021: 3.8 nmol/L)	Not performed	Yes (2022: thymic hyperplasia)	Allergic skin reaction associated with tachycardia and a hypertensive cough, thrombocytopenia, TakoTsubo stress cardiomyopathy (LVEF of 30%), osteoporosis, spondylodiscitis cortisone-induced myopathy, major depressive disorder, and urinary tract infection

* according to the Myasthenia Gravis Foundation of America (MGFA) classification. Abbreviations: AChR, acetylcholine receptor; EMG, electromyography; F, female; gMG, generalized myasthenia gravis; LVEF, left ventricular ejection fraction; M, male; OTI, orotracheal intubation with mechanical ventilation.

## Data Availability

Data sharing is not available due to privacy restrictions.

## Abbreviations

The following abbreviations are used in this manuscript:

AChR	Acetylcholine receptors
ALT	Alanine transaminase
AST	Aspartate transaminase
CBC	Complete blood count
COVID-19	Coronavirus disease 2019
d	Day
D	Dose
EAP	Early Access Program
EMG	Electromyography
F	Female
FcRn	Neonatal Fc receptor
gMG	Generalized myasthenia gravis
IG	Immunoglobulin
IVIg	Intravenous immunoglobulins
IU	International units
LEVF	Left ventricular ejection fraction
M	Male
MAC	Membrane attack complex
MG	Myasthenia gravis
MG-ADL	Myasthenia Gravis Activities of Daily Living
MG-QoL15r	Myasthenia Gravis Quality of Life 15-item revised
MGFA	Myasthenia Gravis Foundation of America
MID	Minimum important difference
NMJ	Neuromuscular junction
OTI	Orotracheal intubation
QMG	Quantitative Myasthenia Gravis
qxd	Every x day(s)
qxh	Every x hour(s)
qxw	Every x week(s)
W	Weeks
